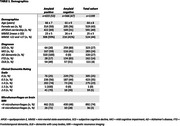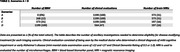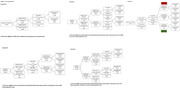# Using a blood‐based biomarker panel for Alzheimer’s disease to determine eligibility for disease modifying treatment in a memory clinic setting: three scenarios

**DOI:** 10.1002/alz.091700

**Published:** 2025-01-09

**Authors:** Sinthujah Vigneswaran, Inge M.W. Verberk, Mariam Gouda, Elsmarieke van de Giessen, David H Wilson, Everard G.B. Vijverberg, Yolande A.L. Pijnenburg, Wiesje M. van der Flier, Charlotte Teunissen, Argonde C. van Harten

**Affiliations:** ^1^ Alzheimer Center Amsterdam, Neurology, Vrije Universiteit Amsterdam, Amsterdam UMC location VUmc, Amsterdam Netherlands; ^2^ Neurochemistry Laboratory, Department of Clinical Chemistry, Amsterdam Neuroscience, Vrije Universiteit Amsterdam, Amsterdam UMC, Amsterdam Netherlands; ^3^ Amsterdam Neuroscience, Neurodegeneration, Amsterdam Netherlands; ^4^ Department of Radiology & Nuclear Medicine, Vrije Universiteit Amsterdam, Amsterdam UMC location VUmc, Amsterdam Netherlands; ^5^ Quanterix, billerica, MA USA

## Abstract

**Background:**

Blood‐based biomarkers (BBM) for Alzheimer’s disease (AD) may be able to identify individuals eligible for emerging anti‐amyloid treatments (DMT). We aimed to evaluate how to use BBM as (pre‐) selection tools for DMTs by simulating different triaging scenarios in a memory clinic setting.

**Methods:**

We included 1199 participants from the Amsterdam Dementia Cohort with measured BBM (plasma pTau181, GFAP and NfL) and used a predefined Youden‐index based cut point for amyloid positivity (method: https://doi.org/10.1002/alz.078748) (Table 1). We created three scenarios by changing at which step in the triaging process BBM were used. Other steps in the triaging process included seeing the medical doctor who determined clinical eligibility and an MRI (Table 1). A traditional screening approach including determination of amyloid positivity by CSF/amyloid‐PET after the MRI was taken as reference scenario A. Scenarios B, C and D all placed BBM determination at different triaging steps (Figure 1). In addition, we tested a scenario E with a high–intermediate–low likelihood categorisation using pre‐defined 90%‐sensitivity and 90%‐specificity levels.

**Results:**

In scenario A, 268 out of 1199 (22%) patients would undergo CSF/amyloid‐PET to select 183 individuals for DMT (Figure 1). When only patients with an abnormal BBM panel were selected to undergo CSF/amyloid‐PET, the number of CSF/amyloid‐PET was reduced with 47% to 141, at the cost of missing 59 (22%) eligible patients. Not performing CSF/amyloid‐PET after BBM measurement in scenario B‐D, resulted in 17 patients receiving treatment without being amyloid positive (12% of all patients receiving DMT) and missing 59 (22% of total eligible) eligible patients. Further consequences for the number of MRIs and medical consultations needed are summarized in Table 2. Only offering CSF/amyloid‐PET to patients with an intermediate chance of being amyloid positive in scenario E would result in 90 CSF/amyloid‐PET evaluations (49%) at the cost of missing 51 (19%) eligible patients and offering DMT to 11 (4%) amyloid negative patients.

**Conclusions:**

Using BBM to preselect patients for DMT may reduce triaging costs, but it comes at the risk of under‐ or over‐treating patients. These unwanted effects can only be reduced by using blood‐based biomarkers with excellent diagnostic value.